# College Dispensary—Professor Fitch’s Clinique before the Class of Rush Medical College

**Published:** 1847-06

**Authors:** 


ARTICLE VII.

### COLLEGE DISPENSARY.

### *Professor Fitch's Clinique.*

[Reported for the Journal.]

*January* 13*th* 1847. Mr. —— presents himself this morning for treatment. We will examine his case. Æt. 35, has been sick since June last; pulse 110, small, and somewhat hard; has considerable thirst; very poor appetite; occasional nausea and vomiting. You perceive his tongue is loaded with a white crust in the centre, while the tip and edges are clear and red. Diarrhœa, with griping pains; sense of tightness and soreness across the abdomen, which is increased by eating and by exertion. His abdomen, you see, is very much tumefied; the tumor elastic, and apparently tympanitic. Has pain across the loins; pricking pains in various parts of the abdomen, and you can readily detect tenderness upon pressure over nearly every part of it. Abdominal pains increased at irregular intervals, without any assignable cause for such increase. A glance at the man shows you that the growth of other parts of his body has been apparently in an inverse ratio with that of his abdomen. His features are sharp and sallow; his extremities emaciated, and the skin upon them flabby. Passes but little and highly coloured urine; is able, and has been most of the time, to walk about and attend to light business, but feels "very weak," and is easily fatigued. I can discover no enlargement of either liver or spleen, though, if the general abdominal tumefaction was less, such enlargements might very probably be detected.

Our diagnosis here must be *Chronic Peritonitis*. We have in his present symptoms, no clue to the cause, though the disease probably had its origin in intermittent fever, of which the patient informs us he had repeated attacks in the autumn of 1845 and the succeeding winter.

The *chronic* occasionally follows *acute* inflammation of the same membrane. More usually, however, it is a primary form of the disease, attended by the *acute*. Insidious in its commencement, and gradual in its progress, it has often so far advanced as to be incurable before its detection. This fact should make you rigid in your examination into cases presenting any of its symptoms; and you must by no means expect always to find them so well marked as in the ease before you. The inflammation may, in its origin, be confined to a limited extent of the membrane; perhaps to that portion of it covering a viscus, as the liver or spleen, and probably accompanied with symptoms which only afford data for assumption, positive or negative, of a co-existent affection of the parenchymatous structure of the subjacent organ. When such affections exist, if it be other than functional, a greater feeling of resistance, fullness, and hardness to the touch, is ordinarily apparent than in the peritoneal inflammation. You will, it is true, find the same hardness, &c., present if the inflammation be of the glandular substance without implication of the covering membrane. But such cases are rare, and when they occur there is not the same degree of soreness—at least the soreness appears deeper seated, requiring greater pressure for its detection, and the pulse is neither so small or rapid. Although the origin of this form of peritoneal inflammation may be thus limited, it rarely remains so through its subsequent stages. On the contrary, if undisturbed and unchecked by appropriate treatment, it continues spreading until it proves fatal by its extent, by some of its terminations, or by interruption or perversion of function which it produces in parts with which it is in immediate relation.

In scrofulous habits, especially in children, you will frequently find that formidable group of symptoms denominated *marasmus*, indicative of an affection of the mesenteric glands, supervening upon this inflammation. The result occasionally attains in the adult. I have seen the intestinal convolutions firmly adherent, so matted together, as to be imperceptible until separated by the knife.

For what disease could the case before us be mistaken? Perhaps most easily for ascites, and the more especially as effusion of serum into the cavity of the abdomen is a frequent consequence of this form of peritonitis. The febrile symptoms found here are common to both, as is the enlargement of the abdomen. In the former, however, you will detect a change of the most prominent part of the enlargement from side to side with the patient's change of position, and fluctuation is distinguishable upon percussion. Here the enlargement is uniform in every position; there is no fluctuation, but the elastic feel and semi-sonorousness of tympanitis. Œdema of the lower extremities is usually present with the former, rarely so with the latter. It might be supposed that diagnosticating the two conditions could not be a matter of very great moment from the fact already mentioned, viz.: ascites being a frequent result of the inflammation. Such a supposition, if practically acted upon would be attended with mischievous consequences. An alterative treatment may be required for ascites, or the removal of its cause. This treatment is the most efficacious for peritoneal inflammation. So far as its adoption is concerned then both cases would be benefitted. We, however, frequently endeavor to remove effused serum, to promote its absorption, and thus relieve the patient of the urgent symptoms dependent upon its quantity, by the administration of hydragogue cathartics. Such treatment would greatly aggravate peritonitis, if it did not ensure a fatal termination; therefore, when we resort to it for removal of serous effusion, consequent upon this disease, we treat for a symptom at the expense of an aggravation of its cause, and can only justify ourselves for so doing by the urgency of the case, and by immediately following it with the adoption of an appropriate radical treatment. Mistaking the case before us for hepatitis, splenitis, or for inflammation of the stomach or intestines, from the tenderness and pain in their respective regions, would be a far less grave error. The parenchyma of the liver or spleen, or the mucous or muscular tissue of the stomach or intestines, may have been the primary seat of the inflammation, but the symptoms now present could not have ensued without its extension to the peritoneum. The treatment anterior and subsequent to such extension would be so nearly identical as to leave but little specially applicable to disease of one or the other tissue. The scant and highly colored urine, with the persistent severe pain across the loins by first fixing the attention of a careless or inattentive observer, might lead to a mistake of the case for one of renal disease. Such mistake could the more readily be made, as the stomach almost uniformly sympathises with diseased kidneys, as shown by the nausea and vomiting; and still farther to increase the liability to such mistake, we find nephralgia often accompanied with an acutely severe pain of the abdomen, midway between the ileum and umbilicus and greatly increased by pressure. If nephralgia was the supposed disease, cathartics and opium would be resorted to, and to an extent which could not fail to be injurious here. If nephritis was imagined to exist, as the treatment would be antiphlogistic, it may be thought the error would not be so important. If you reflect, however, a moment upon the remedies withheld in nephritis, you will perceive that many of them are among the most important in the case before us, as blisters, saline laxatives, and terebinthinates.

A blister will be directed to the patient's epigastrium, to be dressed with ung. hydrarg.; free pustulation with tart. emetic ointment, to be induced over the rest of his abdomen. A powder of—

℞.—Proto-chloride Hydrarg., gr. iij;
Nitrate Potass., gr. v;
Pulv. Doveri, gr. vi; to be taken twice daily until the next report.

*February 4th.*—You will probably recognise Mr. —— as the case of chronic peritonitas before you on the 13th Jan. This is the third report. He has, for two weeks past, been kept mildly but decidedly under mercurial influence. He now tells you his appetite is good, thirst gone, as are the pains and tenderness of the abdomen with which he was formerly tormented. Free pustulation has been kept up over the abdomen, the enlargement of which has mostly disappeared. Pulse 90 and soft. His diarrhœa has been checked, but his bowels yet evince some tendency to laxness. He will now be directed to discontinue the counter irritant, and dress the pustules with simple cerate. The mercurial he was told to discontinue a week since. Bals. copaiva m 20 twice daily will be sufficient to control the remaining evidences of diarrhœa, and perfect the cure. With this he will accordingly be discharged.

